# The effectiveness of virtual reality, augmented reality, and mixed reality training in total hip arthroplasty: a systematic review and meta-analysis

**DOI:** 10.1186/s13018-023-03604-z

**Published:** 2023-02-19

**Authors:** Shilong Su, Ruideng Wang, Rubing Zhou, Zhengyang Chen, Fang Zhou

**Affiliations:** Department of Orthopedics, Peking University Third Hospital, No.49 North Garden Road, Haidian, Beijing, 100191 China

**Keywords:** Augmented reality, Virtual reality, Mixed reality, Training, Total hip arthroplasty, Meta-analysis

## Abstract

**Background:**

Extended reality (XR), including virtual reality (VR), augmented reality (AR), and mixed reality (MR), has been used in the training of total hip arthroplasty (THA). This study aims to examine the effectiveness of XR training in THA.

**Methods:**

In this systematic review and meta-analysis, we searched PubMed (MEDLINE), EMBASE (OVID), Cochrane Central Register of Controlled Trials (CENTRAL), Web of Science, and clinicaltrials.gov from inception to September 2022 for eligible studies. The Review Manager 5.4 software was applied to compare accuracy of inclination and anteversion, and surgical duration between XR training and conventional methods.

**Results:**

We identified 213 articles, of which 4 randomized clinical trials and 1 prospective controlled study including 106 participants met inclusion criteria. The pooled data indicated the XR training had better accuracy of inclination and shorter surgical duration than conventional methods (MD = −2.07, 95% CI [− 4.02 to −0.11], *P* = 0.04; SMD = −1.30, 95% CI [− 2.01 to −0.60], *P* = 0.0003), but the accuracy of anteversion was similar in the two groups.

**Conclusions:**

This systematic review and meta-analysis found XR training had better accuracy of inclination and shorter surgical duration than conventional methods in THA, but the accuracy of anteversion was similar. Based on the pooled results, we suggested that XR training can better improve trainees’ surgical skills than conventional methods in THA.

**Supplementary Information:**

The online version contains supplementary material available at 10.1186/s13018-023-03604-z.

## Introduction

Total hip arthroplasty (THA) is an effective method for the management of severe hip joint disorders [1]. As the incidence of osteoarthritis and other degenerative diseases affecting the bones and joints increases, so does the need for THA [2]. The number of THAs performed annually in the USA is projected to increase from 330,000 in 2010 to 635,000 by 2030 [3], emphasising the importance of prolonging the longevity of implants and reducing the risk of revision through reproducible surgery. Optimal anteversion and inclination angles of the acetabular cup are essential for a successful THA [4–6]. The interaction between acetabular cup position and femoral stem anteversion contributes to the functional range of hip motion, while preventing intra-articular and extra-articular impingements, instability, and polyethylene wear [4–7]. Malposition of the acetabular component increases the risk of dislocation, impingement, pelvic osteolysis, acetabular migration, and acetabular lining wear [8, 9]. Historically, the “safe zone” of acetabular component orientation described by Lewinnek et al. [10] to minimize dislocation risk has been used as a guide; however, the ideal acetabular component position is unknown [11].

THA is a complex operation that generally requires a long learning curve [12, 13]. Low-volume or junior surgeons [14] are more likely to malposition implants outside of the safe zone [10]. Safe, standardized, and effective surgical training for THA is therefore imperative. At present, the commonly used training methods are reading technique manuals, using instruments on low-fidelity synthetic bone models, and observing surgery, and the most realistic environment is the simulation of human cadaver joints, and we call these methods conventional methods. Worldwide, there are reduced surgical training opportunities due to changes in work-hour limits, service reconfiguration, and a focus on patient safety and hospital productivity [15].

Recently, increasing interest has been paid to virtual reality (VR), augmented reality (AR), and new mixed reality (MR), which include both AR and VR, in the medical education world, particularly for anatomy teaching and resident surgical training [16–18]. VR technology generally uses a headset, blocking out visual stimulus from the real world. AR allows users to see the real world, but overlays virtual elements. MR combines the two, including aspects of both the real and virtual world [19, 20]. Extended reality (XR) is the umbrella term that refers to these three different types of technology [21]. Combined with the continued rapid advancements in graphic processor technology and interest from software developers to create immersive surgical skill training simulations, XR users can practice surgical skills in an interactive and risk-free virtual environment. Several studies have shown that XR training was substantially more effective than conventional methods in improving the surgical performance of trainees for THA [22, 23]. XR training resulted in clinically relevant improvements in overall performance, better completion rate of the key surgical steps, more accurate implant position, and faster surgery. But some scholars do not agree with these results [24, 25].

To our knowledge, no systematic review and meta-analysis has explored the effectiveness of XR training in THA. Thus, this systematic review and meta-analysis was designed to compare XR training versus conventional methods in THA in terms of implant positioning, and surgical duration, in order to gain some theoretical insights that may guide clinical practice.

## Methods

We conducted a systematic review and meta-analysis in accordance with the 2020 version of the Preferred Reporting Items for Systematic Reviews and Meta-Analyses (PRISMA 2020) [26]. The study was registered on the International Prospective Register of Systematic Reviews (PROSPERO) (CRD42022366865).

### Search strategy and selection criteria

In an academic medical setting, we searched electronic databases to identify relevant studies from inception to September 2022, including PubMed (MEDLINE), EMBASE (OVID), Cochrane Central Register of Controlled Trials (CENTRAL), Web of Science, and clinicaltrials.gov. Search terms included: (virtual reality OR augmented reality OR mixed reality) AND total hip arthroplasty. To achieve the highest sensitivity, we used a combination of keywords and indexed terms (e.g., PubMed Medical Subject Headings). Complete search algorithms for each database are available in the Additional file 1. We also reviewed the reference lists of eligible studies and previous evidence summaries to identify additional literature. Because our aim was to be as comprehensive as possible in the systematic review, we did not place time or publication status limits to the search except for restriction to the English language.

The criteria for inclusion were research articles studying virtual reality, augmented reality, and mixed reality training compared to conventional methods in THA and reporting on the accuracy of implant placement and surgical duration. Studies were excluded if they were technical notes, letters to the editor, expert opinions, review articles, meta-analyses, scientific conference abstracts, and case reports. Our primary search objective addressed the PICO (population, intervention, comparator and outcomes) question. The PICO criteria for study inclusion are as shown in Table 1. Two reviewers (SS and RW) working independently considered the potential eligibility of each of the abstracts generated by the search strategy. The combination of EndNote software (version X9, Thomson Corporation, Stanford, USA) and manual screening was carried out to remove duplicates. Full-text articles were obtained unless both reviewers decided that an abstract was ineligible. Each full-text report was assessed independently for final study inclusion. Disagreement was resolved through discussion and consensus between the reviewers (SS and RW).

**Table 1:** ‘Population, Intervention, Comparator, Outcomes’ (PICO) criteria for eligibility

	PICO criteria
Population	Medical trainees ranging from medical students to consultant level
Intervention	Virtual reality, augmented reality, and mixed reality training in total hip arthroplasty
Comparator	Conventional methods training in total hip arthroplasty including reading technique manuals, using instruments on low-fidelity synthetic bone models, and observing surgery, and the simulation of human cadaver joints
Outcomes	The accuracy of inclination and anteversion, and surgical duration

### Data extraction and quality assessment

Two reviewers (SS and RW) independently extracted data from each article using a predefined data extraction form. Any disagreements between them were solved by a discussion (SS and RW). The included studies were evaluated for the authors, year of publication, country, study design, the type of XR, details of learners, the accuracy of inclination and anteversion, and surgical duration. In addition, the main conclusion from each study was also recorded. If data were not presented in the original article, corresponding authors were contacted to acquire the missing data, although no responses were received.

Two reviewers (SS and RW) evaluated the quality of included studies independently, utilizing the version 2 of the Risk of Bias tool of the Cochrane Library (RoB 2) [27]. The risk of bias tool comprised seven elements from five domains (selection, reporting, performance, attrition, and other), and each of these items can be judged as low, unclear, and high risk. Disagreement was resolved through discussion and consensus between the reviewers (SS and RW).

### Statistical analysis

Data were pooled and analyzed using the Review Manager software (version 5.4, Cochrane Collaboration, Oxford, UK). A *P*-value of < 0.05 was considered significant. For continuous data, the mean difference (MD) along with the 95% confidence interval (CI) was used to perform analysis, including the accuracy of inclination and anteversion. If different measurement methods or units were used for the same index and the mean values were significantly different, standardized mean difference (SMD) with 95% CI was used, such as surgical duration. One study [28] used different methods to measure the surgical duration. *I*^2^ statistic was used to assess the heterogeneity, and significant heterogeneity was identified as *I*^2^ > 50% and *P* < 0.10. The random-effect or fixed-effect model was selected depending on the heterogeneity of the included studies. When significant heterogeneity (*I*^2^ > 50%; *P* < 0.10) occurred, random-effect model was used and if not, a fixed-effect model was selected. In case of significant heterogeneity, sensitivity analysis was conducted to evaluate the influence of every single study on the meta-analysis outcome by excluding studies one by one. Forest plots were used to present the results of the individual studies and respective pooled estimates of effect size. Publication bias was estimated by a funnel plot and a publication bias was considered present if an asymmetry in the funnel plot was found. According to Egger et al. [29], the ability of funnel plots to detect this bias is limited when the number of included studies is small. Consequently, we ensure to provide the funnel plot as a supplementary file.

## Results

### Search findings

**Fig. 1:**
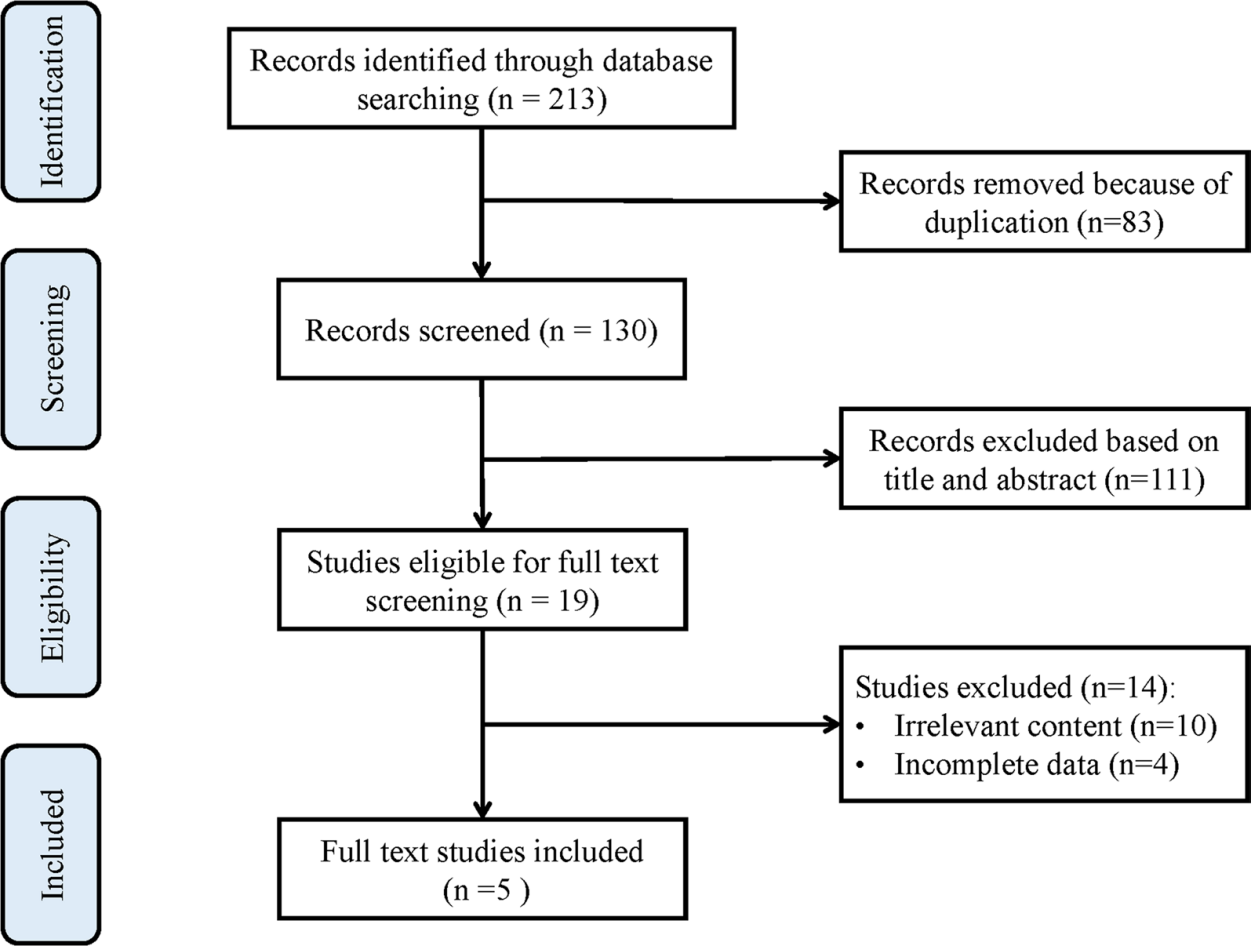
PRISMA (Preferred Reporting Items for Systemic Meta-Analyses) study selection flow diagram

A total of 213 potentially relevant articles were extracted from the 5 electronic databases (Additional file 2). After deleting 83 duplicates, 111 irrelevant articles were excluded after their titles and abstracts were reviewed. We reviewed the remaining 19 full-text articles. We excluded another 14 articles for irrelevant content or incomplete data. Ultimately, 5 studies were included in this systematic review and meta-analysis. The detailed study selection process is shown in Fig. 1.

### Study characteristics and quality assessment

Overall, 5 studies, published from 2018 to 2020, met the inclusion requirements, including four randomized clinical trials (RCTs) [22, 24, 28, 30], and one prospective controlled study [31]. These studies involved 106 participants: 24 were medical students, 78 were residents, and 4 were expert hip surgeons. There were two studies performed in the USA and three studies in the UK. The types of XR used included VR and AR, without MR; conventional methods included traditional learning (lectures, textbooks, and videos), standard fluoroscopic technique, and training by an expert surgeon. Table 2 shows the characteristics of included studies.

**Table 2:** Characteristics of included studies

Author	Country	Study Design	Participants	N (XR/control)	Surgical approach	Surgical conditions	Type of XR	Comparator	Accuracy of inclination		Accuracy of anteversion		Surgical duration	
									XR	Control	XR	Control	XR	Control
Kartik et al.,2018 [24]	UK	RCT	Medical students	12/12	Posterior	Phantom hip model	AR	Training by an expert surgeon	NA	NA	NA	NA	NA	NA
Kartik et al.,2019 [30]	UK	RCT	Residents	12/12	Direct anterior	Whole-body cadaver	VR	THA operation manual and annotated videos	3° ± 3°	15° ± 8°	4° ± 3°	16° ± 6°	42 ± 7 min	51 ± 9 min
Jessica et al.,2019 [22]	USA	RCT	Residents	7/7	Standard posterolateral	Pelvis-to-toes cadaver specimen	VR	Standard study materials	NA	NA	NA	NA	NA	NA
Kartik et al.,2020 [31]	UK	Prospective controlled study	Residents and expert hip surgeons	32/4	Direct anterior	Dry bone hip model or VR platform	VR	Expert hip surgeons	4° ± 2°	5° ± 2°	6° ± 3°	4° ± 2°	36 ± 6 min	30 ± 5 min
Clayton et al., 2020 [28]	USA	RCT	Residents	8/8	Direct anterior	Radiopaque foam pelvis	AR	Standard Fluoroscopic Technique	1.8° ± 1.4°	4.8° ± 2.2°	1.4° ± 0.7°	4.8° ± 3.2°	1.8 ± 0.25 min	3.9 ± 1.6 min

N, the number of participants; XR, extended reality; RCT, randomized clinical trial; AR, augmented reality, NA, not available; VR, virtual reality; THA, total hip arthroplasty

**Fig. 2:**
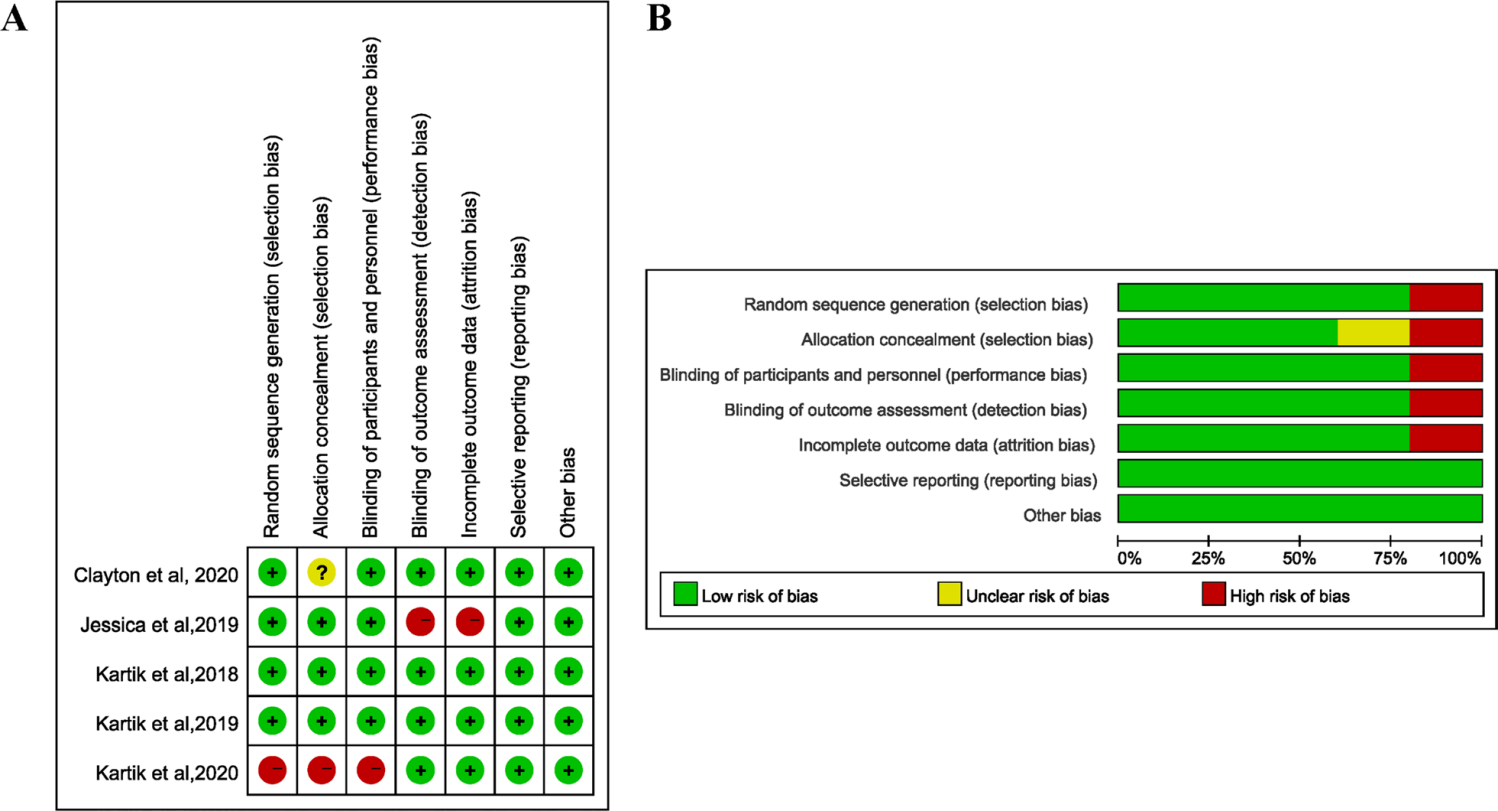
Quality evaluation of included studies utilizing the Risk of Bias tool of the Cochrane Library. **A** Judgements of authors about each risk of bias item for each included study, **B** about each risk of bias item presented as percentages across all included studies

The result of the quality assessment was summarized in Fig. 2. The risk of bias was low for most of the domains. One study was rated to have high risk due to the absence of randomization, allocation concealment, and blinding [31]. Blinding of outcome assessment and incomplete outcome data in another study were reported high risk of bias [22].

### Accuracy of inclination and anteversion

**Fig. 3:**
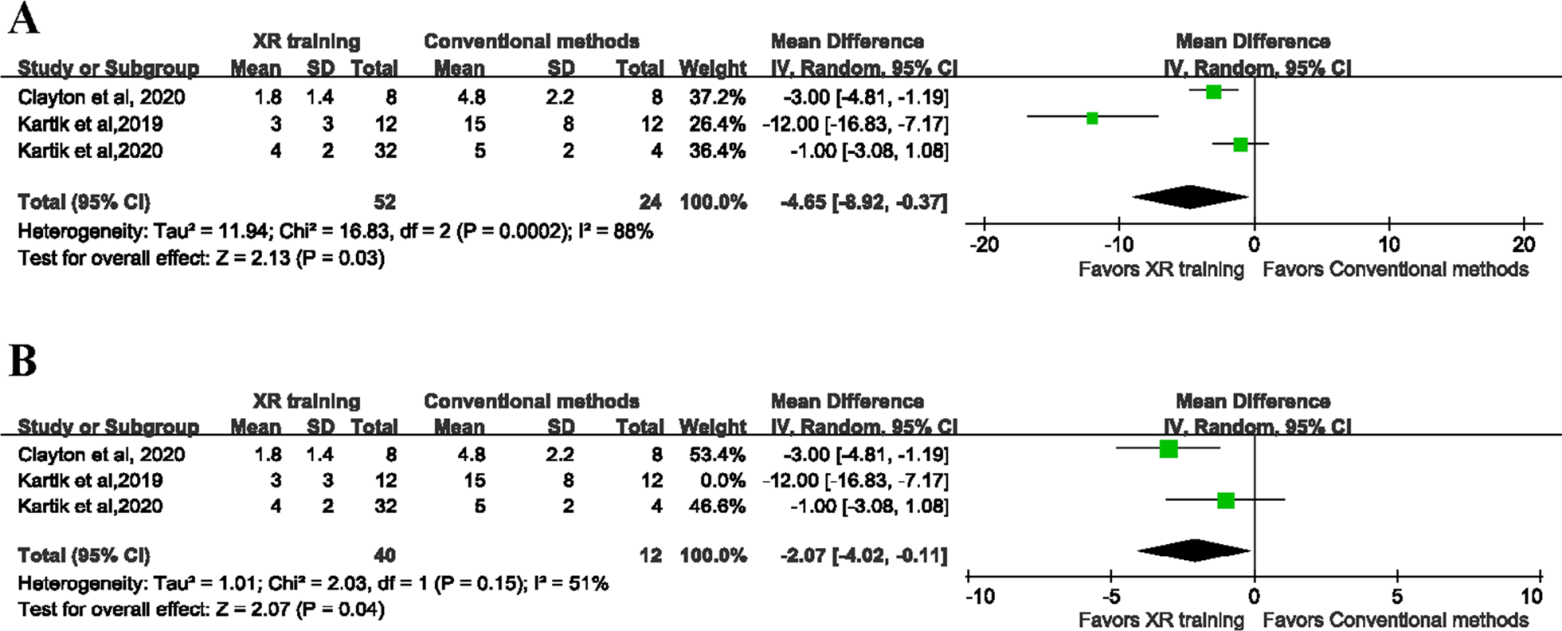
**A** Forest plot to assess accuracy of inclination between two groups. B. Forest plot to assess accuracy of inclination after removing one study between two groups

**Fig. 4:**
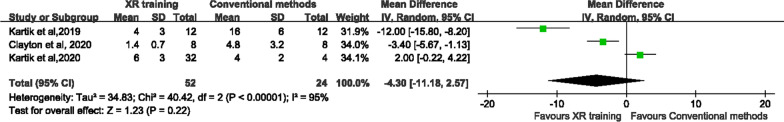
Forest plot to assess accuracy of anteversion between two groups

Three of the included studies involving 68 participants reported the accuracy of inclination [28, 30, 31]. The random effects model was employed in the meta-analysis because of significant heterogeneity between the studies (*P* = 0.0002, *I*^2^ = 88%). The results showed that the XR training had better accuracy of inclination than conventional methods (MD = −4.65, 95% CI [− 8.92 to −0.37], *P* = 0.03) (Fig. 3A). When performing the sensitivity analysis, the heterogeneity came from one study [30]. As such, we performed analysis again after removing the study, and the pooled results also showed that the XR training had better accuracy of inclination than conventional methods (MD = −2.07, 95% CI [− 4.02 to −0.11], *P* = 0.04) (Fig. 3B). Also, three studies with 68 participants provided the accuracy of anteversion for analysis [28, 30, 31]. Because the data was significantly heterogeneous (*P* < 0.00001, *I*^2^ = 95%), the data analysis was performed with a random-effect model, showing no significant difference between the two groups (MD = −4.30, 95% CI [− 11.18 to 2.57], *P* = 0.22). The pooled result was shown in Fig. 4. We found that heterogeneity was still high after excluding studies one by one. We did not find the source of heterogeneity after intensive reading of three studies. One study [24] does not directly show the accuracy of inclination and anteversion, but uses orientation error to express. Orientation error is derived from the Pythagorean calculation by inclination error and anteversion error. There were no differences in orientation error between the expert surgeon-trained group and the AR group on the test using concealed pelvic tilt (6° ± 4°versus 7° ± 5°, *P* = 0.301).

### Surgical duration

**Fig. 5:**
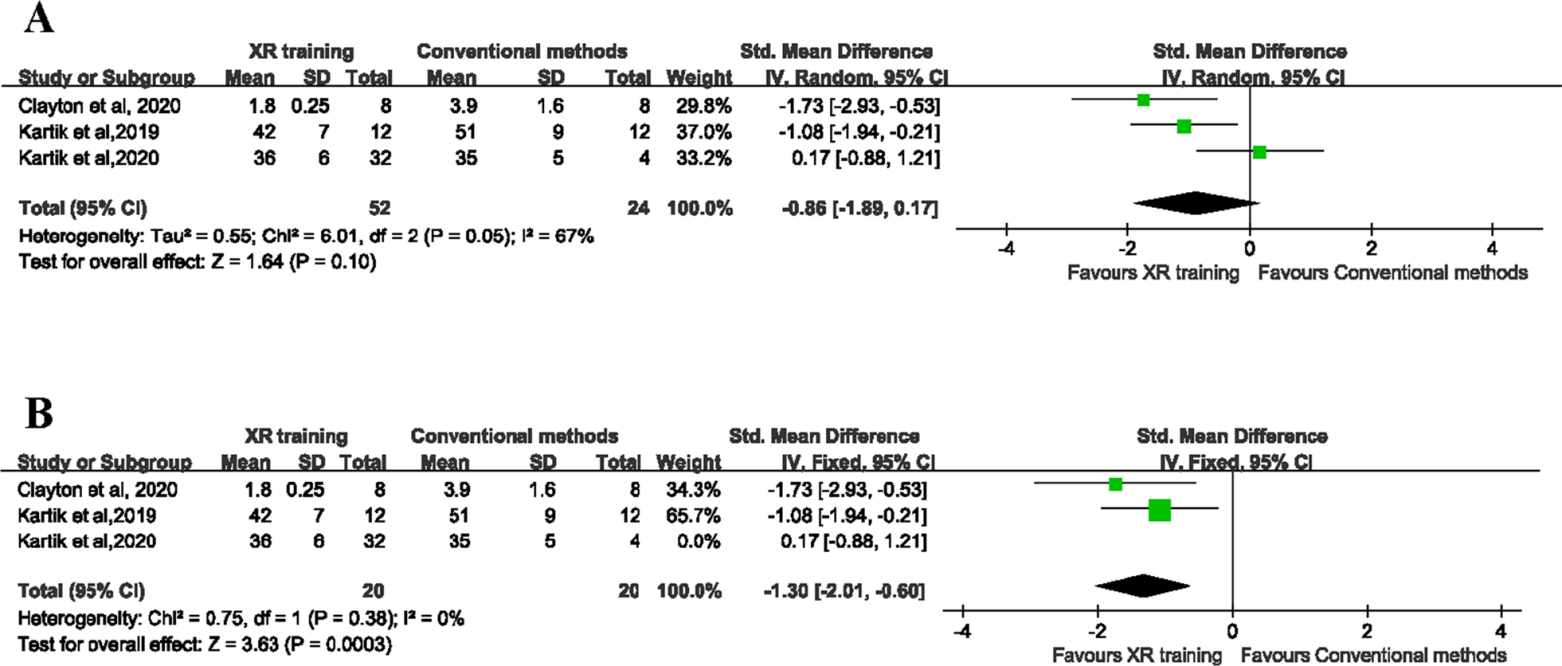
**A** Forest plot to assess surgical duration between two groups. **B** Forest plot to assess surgical duration after removing one study between two groups

Three of the included studies involving 68 participants reported the surgical duration [28, 30, 31]. Since the heterogeneity between the studies was significant (*P* = 0.05, *I*^2^ = 67%), the random effects model was used for the meta-analysis. The results showed that the surgical duration was similar in the two groups (SMD = −0.86, 95% CI [− 1.89 to 0.17], *P* = 0.10), and the difference was not statistically significant (Fig. 5A). Because of the existence of significant heterogeneity, the sensitivity analysis was performed and indicated that the heterogeneity come from one study [31]. Therefore, we performed analysis again after removing the study; however, the pooled results (Fig. 5B) revealed the XR training had shorter surgical duration than conventional methods (SMD = −1.30, 95% CI [− 2.01 to −0.60], *P* = 0.0003).

Here is a RCT [22], whose primary outcomes were improvement in test and cadaver THA scores, without directly measuring inclination, anteversion, or surgical duration. We included it in the review and found that VR training improves residents’ surgical skills but has no significant effect on medical knowledge between the VR and control groups. The most significant improvement was seen in technical skills.

### Publication bias

To address publication bias, we created funnel plots for all analyses. The funnel plots of the accuracy of inclination and anteversion revealed asymmetry, indicating evidence of publication bias (Additional file 3). However, only three studies were included in the funnel plots; therefore, this result should be interpreted with caution.

## Discussion

The principal finding of this systematic review and meta-analysis was that the XR training had better accuracy of inclination and shorter surgical duration than conventional methods in THA, but the accuracy of anteversion was similar in the two groups. One study [24] showed there were no differences in orientation error between the expert surgeon-trained group and the AR group. But orientation error is derived from the Pythagorean calculation by inclination error and anteversion error. Similar orientation error may be obtained by calculation of different inclination error and similar anteversion error. The results of this study may be consistent with our results. Unfortunately, we contacted the corresponding author to get the original data and did not get a response. To our knowledge, this is the first systematic review and meta-analysis to show the effectiveness of virtual reality, augmented reality, and mixed reality training in THA.

Surgery is a technical and learned skill, and reaching the expert level in surgery requires complex skills such as advanced pattern recognition, self-monitoring, and minimizing distractions [32]. If we think that about 75% of the important events in surgery are related to decision-making and 25% are related to dexterity, no simulation can replace the real surgical environment as a place to learn real surgical essentials from surgeons [22]. However, the operating room may not always be an ideal place to learn surgical skills, because of many problems related to patient safety, strict time limits, and so on [33]. Surgical technique training is a necessary process for real surgery in the operating room. Current orthopedic simulators cost approximately USD 100,000 [34], and most have yet to be validated to demonstrate the acquisition of skills by learners. The phantom hip and AR platform developed here cost approximately USD 10,000 [24]. Although this figure does not include institutional costs for software development, the platform is less expensive than currently available arthroscopic and open surgical platforms.

In addition, in the process of learning complex procedures, the attention of surgeons is always limited: early learners focus on learning technical skills and coordination of their movement, while training experts may demonstrate more intraoperative decision-making, planning, and avoiding adverse events based on their clinical experience [35]. This cannot be realized in the existing simulator. XR simulation can help early learners move toward automation outside the operating room as a supplement to conventional training, and help them focus on learning complex behaviors during the intraoperative experience [31]. Several studies in the field of general surgery have shown that skills learned in VR simulations can improve the performance of residents in the operating room [36, 37]. Also, VR and AR simulators have been validated to develop technical expertise for orthopedic fields, including drilling bone [38], and applying a femoral plate for fracture fixation [39]. As in our study, these have demonstrated that similar unsupervised training improves performance in novices. XR training can help trainees become familiar with the three-dimensional anatomy and instrumentation used to perform the operation without the labpratory space and instrument trays required for traditional simulation. Compared to learning from a video, XR simulation provides a more active learning experience. Although XR simulation does not provide real haptic feedback to trainees, it can vividly teach the flow and steps of surgery through pre-set procedural steps, which will be beneficial in the operating room environment.

At present, VR and AR simulators can help students to perform better [38–40]. But the data collection capabilities of them are limited. In the study, we did not find a training simulator based on MR. MR technology is a new type of digital holographic imaging technology that combines the advantages of VR and AR technology. By introducing real scene information into the virtual environment, it sets up an interactive feedback information loop between the virtual world, the real world, and users, to enhance the reality of user experience. Its key is to interact with the real world and obtain information promptly and to interact seamlessly with users of the real world and virtual models [20]. Perhaps in the near future, the development of MR training software can provide us with training in more complex surgical scenarios, which is suitable for higher-level surgeons. For example, it may be helpful to simulate periprosthetic fractures, revision hip arthroplasty, or hip dysplasia cases. Moreover, building decision-making questions into the software may help trainees learn more than procedural steps from the simulation.

The present study has some limitations. Firstly, there were only 5 studies included in this study with a total of 106 participants. The sample size of our study is small, which weakens the reliability of the study conclusion. Secondly, both RCTs and non-RCTs were included in our analysis due to lack of data, which adds to potential bias to this study. Thirdly, some of the studies did not contain sufficient information for pooled analyses. Although we tried to contact the authors for raw data, we were unable to do so. Finally, the language bias was inevitable since all included studies were published in English.

## Conclusion

This systematic review and meta-analysis found XR training had better accuracy of inclination and shorter surgical duration than conventional methods in THA, but the accuracy of anteversion was similar. Based on the pooled results, we suggested that XR training can better improve trainees’ surgical skills than conventional methods in THA.

## Supplementary Information

**Additional file 1.** Database Search Algorithms.

**Additional file 2.** 213 potentially relevant articles.

**Additional file 3.** Funnel plots to assess publication bias on the accuracy of inclination and anteversion.

## Data Availability

The datasets used and/or analyzed during the current study are available from the corresponding author on reasonable request.

## Abbreviations

XR: Extended reality
VR: Virtual reality
AR: Augmented reality
MR: Mixed reality
THA: Total hip arthroplasty
PRISMA: Preferred Reporting Items for Systematic Reviews and Meta-Analyses
MD: Mean difference
OR: Odds ratio
CI: Confidence interval
RCT: Randomized clinical trials
