# Supplementary material for: The effectiveness of virtual reality, augmented reality, and mixed reality training in total hip arthroplasty: a systematic review and meta-analysis
Source: J Orthop Surg Res. 2023 Feb 19;18:121. doi: 10.1186/s13018-023-03604-z (PMC9940416; doi:10.1186/s13018-023-03604-z)
Supplement: Supplementary file 2 — Additional file 2. 213 potentially relevant articles. [file 13018_2023_3604_MOESM2_ESM.docx]

**213 potentially relevant articles**

1. Zhao W, Yang S, Luo X. Towards Rehabilitation at Home After Total Knee Replacement. Tsinghua Science and Technology. 2021;26(6):791-9.
2. Zhao J-X, Su X-Y, Zhao Z, Xiao R-X, Zhang L-C, Tang P-F. Radiographic assessment of the cup orientation after total hip arthroplasty: a literature review. Annals of Translational Medicine. 2020;8(4).
3. Zhao JX, Su XY, Zhao Z, Xiao RX, Zhang LC, Tang PF. Radiographic assessment of the cup orientation after total hip arthroplasty: a literature review. Ann Transl Med. 2020;8(4):130.
4. Zhang Y, Wang X, Shao Y, Xia Q. The orientation of the surgical epicondylar axis varies in varus and non-varus knees in the coronal plane. Knee Surgery Sports Traumatology Arthroscopy. 2018;26(9):2580-6.
5. Zhang X, Xiao H, Chen Y. Evaluation of a WeChat‐based life review programme for cancer patients: a quasi‐experimental study. Journal of advanced nursing (john wiley & sons, inc). 2019;75(7):1563‐74.
6. Zhang R, Shen J, Liu Q, Qi Y, Wu X, Cai S, et al. AUGMENTED REALITY NAVIGATION FRAMEWORK FOR TOTAL HIP ARTHROPLASTY SURGERY. Journal of Mechanics in Medicine and Biology. 2021;21(08).
7. Zavala-González J, Martínez D, Gutiérrez-Espinoza H. Effectiveness of adding virtual reality to physiotherapeutic treatment in patients with total hip arthroplasty. A randomized controlled trial. Clin Rehabil. 2022;36(5):660-8.
8. Zavala-González J, Martínez D, Gutiérrez-Espinoza H. Effectiveness of adding virtual reality to physiotherapeutic treatment in patients with total hip arthroplasty. A randomized controlled trial. Clinical rehabilitation. 2022;36(5):660‐8.
9. Zavala-González J, Martínez D, Gutiérrez-Espinoza H. Effectiveness of adding virtual reality to physiotherapeutic treatment in patients with total hip arthroplasty. A randomized controlled trial. Clinical rehabilitation. 2022;36(5):660-8.
10. Zavala-Gonzalez J, Martinez D, Gutierrez-Espinoza H. Effectiveness of adding virtual reality to physiotherapeutic treatment in patients with total hip arthroplasty. A randomized controlled trial. Clinical Rehabilitation. 2022;36(5):660-8.
11. Zannoni C, Viceconti M, Lattanzi R, Petrone M, editors. Efficacy of fully 3D monomodal interface in pre-operative planning of total hip replacement. 17th International Congress and Exhibition of Computer Assisted Radiology and Surgery; 2003 2003 Jun 25-28; London, England2003.
12. Yang J, Guo J, Tang Y, Huang L, Wiley J, Zhou Z, et al. The mediating effect of coping styles and self‐efficacy between perceived stress and satisfaction with QOL in Chinese adolescents with type 1 diabetes. Journal of advanced nursing (john wiley & sons, inc). 2019;75(7):1439‐49.
13. y6cz RBR. Virtual Reality in diabetic elderly. https://trialsearchwhoint/Trial2aspx?TrialID=RBR-67y6cz. 2019.
14. Xu Z, Zhang Y. What's new in artificially intelligent joint surgery in China? The minutes of the 2021 IEEE ICRA and literature review. Arthroplasty. 2022;4(1).
15. Xu Z, Zhang H, Wei W, Yang Z. Virtual Scene Construction for Seismic Damage of Building Ceilings and Furniture. Applied Sciences-Basel. 2019;9(17).
16. Xu Z, Zhang H, Lu X, Xu Y, Zhang Z, Li Y. A prediction method of building seismic loss based on BIM and FEMA P-58. Automation in Construction. 2019;102:245-57.
17. Xin Zhao J, Yun Su X, Zhao Z, Xiu Xiao R, Cheng Zhang L, Fu Tang P. Radiographic assessment of the cup orientation after total hip arthroplasty: A literature review. Annals of Translational Medicine. 2020;8(4).
18. Wiese A, Williams G, Lecakes G, Morley M, Kim TW, Almon A, et al., editors. Virtual Medical Instruments for Orthopedic Surgery Training: A Hip Arthroplasty Application. IEEE Sensors Applications Symposium (SAS); 2021 2021 Aug 23-25; Electr Network2021.
19. Waterson HB, Clement ND, Eyres KS, Mandalia VI, Toms AD. The early outcome of kinematic versus mechanical alignment in total knee arthroplasty. Bone & Joint Journal. 2016;98B(10):1360-8.
20. Wang X, Hunter DJ, Vesentini G, Pozzobon D, Ferreira ML. Technology-assisted rehabilitation following total knee or hip replacement for people with osteoarthritis: a systematic review and meta-analysis. Bmc Musculoskeletal Disorders. 2019;20(1).
21. Wang M, Li D, Shang X, Wang J. A review of computer-assisted orthopaedic surgery systems. International Journal of Medical Robotics and Computer Assisted Surgery. 2020;16(5).
22. Wang L, Thoreson AR, Trousdale RT, Morrey BF, Dai K, An K-N. Radiographic cup anteversion measurement corrected from pelvic tilt. Medical Engineering & Physics. 2017;49:103-8.
23. Viceconti M, Testi D, Toni A. Computer-assisted three-dimensional preoperative planning in hip surgery. Minerva Ortopedica e Traumatologica. 2005;56(2):81-92.
24. Vaughan N, Dubey VN, Wainwright TW, Middleton RG. A review of virtual reality based training simulators for orthopaedic surgery. Medical Engineering & Physics. 2016;38(2):59-71.
25. Umin. Randomized controlled study comparing Augmented Reality (AR) Hip Navigation System versus conventional instrumentation on THA in Lateral Decubitus position. https://trialsearchwhoint/Trial2aspx?TrialID=JPRN-UMIN000047608. 2022.
26. Umin. Acetabular cup orientation using portable navigation system applied to total hip arthroplasty in the lateral position: augmented reality-based versus accelerometer-based navigation system A prospective randomized controlled trial. https://trialsearchwhoint/Trial2aspx?TrialID=JPRN-UMIN000045218. 2021.
27. Umin. A pilot study of a simple navigation system for artificial hip joint surgery using augmented reality technology. https://trialsearchwhoint/Trial2aspx?TrialID=JPRN-UMIN000030760. 2018.
28. Umin. A pilot study of a simple navigation system for Total Hip Arthroplasty in the lateral position using augmented reality technology. https://trialsearchwhoint/Trial2aspx?TrialID=JPRN-UMIN000031602. 2018.
29. Tsukada S, Ogawa H, Hirasawa N, Nishino M, Aoyama H, Kurosaka K. Augmented Reality- vs Accelerometer-Based Portable Navigation System to Improve the Accuracy of Acetabular Cup Placement During Total Hip Arthroplasty in the Lateral Decubitus Position. J Arthroplasty. 2022;37(3):488-94.
30. Tsukada S, Ogawa H, Hirasawa N, Nishino M, Aoyama H, Kurosaka K. Augmented Reality- vs Accelerometer-Based Portable Navigation System to Improve the Accuracy of Acetabular Cup Placement During Total Hip Arthroplasty in the Lateral Decubitus Position. Journal of Arthroplasty. 2022;37(3):488-94.
31. Tsai MD, Hsieh MS. Volume manipulations for simulating bone and joint surgery. Ieee Transactions on Information Technology in Biomedicine. 2005;9(1):139-49.
32. Tovihoudji PG, Akponikpe PBI, Agbossou EK, Bertin P, Bielders CL. Fertilizer microdosing enhances maize yields but may exacerbate nutrient mining in maize cropping systems in northern Benin. Field Crops Research. 2017;213:130-42.
33. Testi D, Zannoni C, Petrone M, Clapworthy GJ, Nieberg D, Tsagarakis NG, et al., editors. A multimodal and multisensorial pre-operative planning environment for total hip replacement. 3rd International Conference on Medical Information Visualisation - BioMedical Visualisation; 2005 2005 Jul 05-07; London, ENGLAND2005.
34. Testi D, Lattanzi R, Benvegnù M, Petrone M, Zannoni C, Viceconti M, et al. Efficacy of stereoscopic visualization and six degreesof freedom interaction in preoperative planningof total hip replacement. Medical Informatics and the Internet in Medicine. 2006;31(3):205-18.
35. Testi D, Lattanzi R, Benvegnu M, Petrone M, Zannoni C, Viceconti M, et al. Efficacy of stereoscopic visualization and six degrees of freedom interaction in preoperative planning of total hip replacement. Medical Informatics and the Internet in Medicine. 2006;31(3):205-18.
36. Tanji A, Nagura T, Iwamoto T, Matsumura N, Nakamura M, Matsumoto M, et al. Total elbow arthroplasty using an augmented reality-assisted surgical technique. Journal of Shoulder and Elbow Surgery. 2022;31(1):175-84.
37. Sugano N, Sasama T, Nishihara S, Nakase H, Nishii T, Miki H, et al., editors. Clinical applications of a laser guidance system with dual laser beam rays as augmented reality of surgical navigation. 16th International Congress and Exhibition on Computer Assisted Radiology and Surgery; 2002 2002 Jun 26-29; Paris, France2002.
38. Sugand K, Wescott RA, Carrington R, Hart A, van Duren BH. Training and Transfer Effect of FluoroSim, an Augmented Reality Fluoroscopic Simulator for Dynamic Hip Screw Guidewire Insertion: a Single-Blinded Randomized Controlled Trial. Journal of bone and joint surgery American volume. 2019;101(17):e88.
39. Sugand K, Wescott RA, Carrington R, Hart A, van Duren BH. Training and Transfer Effect of FluoroSim, an Augmented Reality Fluoroscopic Simulator for Dynamic Hip Screw Guidewire Insertion A Single-Blinded Randomized Controlled Trial. Journal of Bone and Joint Surgery-American Volume. 2019;101(17).
40. Su S, Lei P, Wang C, Gao F, Zhong D, Hu Y. Mixed Reality Technology in Total Knee Arthroplasty: An Updated Review With a Preliminary Case Report. Frontiers in Surgery. 2022;9.
41. Su C-H, Cheng C-H. Developing and Evaluating Creativity Gamification Rehabilitation System: The Application of PCA-ANFIS Based Emotions Model. Eurasia Journal of Mathematics Science and Technology Education. 2016;12(5):1443-68.
42. Shah NV, Gold R, Dar Q-A, Diebo BG, Paulino CB, Naziri Q. Smart Technology and Orthopaedic Surgery: Current Concepts Regarding the Impact of Smartphones and Wearable Technology on Our Patients and Practice. Current Reviews in Musculoskeletal Medicine. 2021;14(6):378-91.
43. Schnurr C, Beckmann J, Luering C, Tibesku C, Schlueter-Brust KU, Ettinger M, et al. Status and future of modern technologies in arthroplasty Results of a survey of the German Society for Orthopedics and Trauma Surgery (DGOU). Orthopadie. 2022;51(9):757-62.
44. Schega L, Hamacher D, Wagenaar RC. A comparison of effects of augmented reality and verbal information based interventions in elderly women after hip replacement. Archives of physical medicine and rehabilitation. 2011;92(10):1734‐5.
45. Rossi SMP, Mancino F, Sangaletti R, Perticarini L, Lucenti L, Benazzo F. Augmented Reality in Orthopedic Surgery and Its Application in Total Joint Arthroplasty: A Systematic Review. Applied Sciences-Basel. 2022;12(10).
46. Rodriguez YBF, Liu H. Letter to the Editor on "Augmented Reality Based Navigation for Computer Assisted Hip Resurfacing: A Proof of Concept Study". Annals of biomedical engineering. 2019;47(11):2154.
47. Rodriguez y Baena F, Liu H. Letter to the Editor on "Augmented Reality Based Navigation for Computer Assisted Hip Resurfacing: A Proof of Concept Study". Annals of biomedical engineering. 2019;47(11):2154-.
48. Robb H, Scrimgeour G, Boshier P, Przedlacka A, Balyasnikova S, Brown G, et al. The current and possible future role of 3D modelling within oesophagogastric surgery: a scoping review. Surgical Endoscopy and Other Interventional Techniques. 2022;36(8):5907-20.
49. Rittmeister M, Eisenbeis K, Hanusek S, Yanik-Karaca Z, Starker M, Arabmotlagh M. Assessment of implant position of CTX-custom-made stems with EBRA-FCA in 107 cases of total joint replacement. Zeitschrift Fur Orthopadie Und Ihre Grenzgebiete. 2004;142(6):659-65.
50. Putzer D, Klug S, Moctezuma JL, Nogler M. The use of time-of-flight camera for navigating robots in computer-aided surgery: monitoring the soft tissue envelope of minimally invasive hip approach in a cadaver study. Surg Innov. 2014;21(6):630-6.
51. Putzer D, Klug S, Moctezuma JL, Nogler M. The Use of Time-of-Flight Camera for Navigating Robots in Computer-Aided Surgery: Monitoring the Soft Tissue Envelope of Minimally Invasive Hip Approach in a Cadaver Study. Surgical Innovation. 2014;21(6):630-6.
52. Ponce BA. CORR Insights®: Does An Augmented Reality-based Portable Navigation System Improve the Accuracy of Acetabular Component Orientation During THA? A Randomized Controlled Trial. Clin Orthop Relat Res. 2020;478(5):944-6.
53. Ponce BA. CORR InsightsÂ®: does An Augmented Reality-based Portable Navigation System Improve the Accuracy of Acetabular Component Orientation During THA? A Randomized Controlled Trial. Clinical orthopaedics and related research. 2020.
54. Ponce BA. CORR Insights (R): Does An Augmented Reality-based Portable Navigation System Improve the Accuracy of Acetabular Component Orientation During THA? A Randomized Controlled Trial. Clinical Orthopaedics and Related Research. 2020;478(5):944-6.
55. Ponce BA. CORR Insights®: Does An Augmented Reality-based Portable Navigation System Improve the Accuracy of Acetabular Component Orientation during THA? A Randomized Controlled Trial. Clinical Orthopaedics and Related Research. 2020;478(5):944-6.
56. Pflugi S, Vasireddy R, Lerch T, Ecker TM, Tannast M, Boemke N, et al., editors. Augmented Marker Tracking for Peri-acetabular Osteotomy Surgery. 39th Annual International Conference of the IEEE-Engineering-in-Medicine-and-Biology-Society (EMBC); 2017 2017Jul 11-15; South Korea2017.
57. Petrolo L, Testi D, Taddei F, Viceconti M. Effect of a virtual reality interface on the learning curve and on the accuracy of a surgical planner for total hip replacement. Computer methods and programs in biomedicine. 2010;97(1):86-91.
58. Petrolo L, Testi D, Taddei F, Viceconti M. Effect of a virtual reality interface on the learning curve and on the accuracy of a surgical planner for total hip replacement. Computer methods and programs in biomedicine. 2010;97(1):86-91.
59. Petrolo L, Testi D, Taddei F, Viceconti M. Effect of a virtual reality interface on the learning curve and on the accuracy of a surgical planner for total hip replacement. Computer methods and programs in biomedicine. 2010;97(1):86-91.
60. Peng MJ-Q, Chen H-Y, Hu Y, Ju X, Bai B. Finite Element Analysis of porously punched prosthetic short stem virtually designed for simulative uncemented Hip Arthroplasty. Bmc Musculoskeletal Disorders. 2017;18.
61. Peng MJ, Chen HY, Hu Y, Ju X, Bai B. Finite Element Analysis of porously punched prosthetic short stem virtually designed for simulative uncemented Hip Arthroplasty. BMC Musculoskelet Disord. 2017;18(1):295.
62. Pelliccia L, Lorenz M, Heyde C-E, Kaluschke M, Klimant P, Knopp S, et al. A cadaver-based biomechanical model of acetabulum reaming for surgical virtual reality training simulators. Scientific Reports. 2020;10(1).
63. Pelliccia L, Lorenz M, Heyde CE, Kaluschke M, Klimant P, Knopp S, et al. A cadaver-based biomechanical model of acetabulum reaming for surgical virtual reality training simulators. Sci Rep. 2020;10(1):14545.
64. Pelliccia L, Lorenz M, Heyde CE, Kaluschke M, Klimant P, Knopp S, et al. A cadaver-based biomechanical model of acetabulum reaming for surgical virtual reality training simulators. Scientific reports. 2020;10(1):14545.
65. Park A, Duncan ST, Nunley RM, Keeney JA, Barrack RL, Nam D. Relationship of the posterior femoral axis of the "kinematically aligned" total knee arthroplasty to the posterior condylar, transepicondylar, and anteroposterior femoral axes. Knee. 2014;21(6):1120-3.
66. Otake Y, Suzuki N, Hattori A, Miki H, Yamamura M, Yonenobu K, et al., editors. Hip Motion Analysis Using Multi Phase (Virtual and Physical) Simulation of the Patient-specific Hip Joint Dynamics. 16th Conference on Medicine Meets Virtual Reality; 2008 2008Jan 30-Feb 01; Long Beach, CA2008.
67. Otake Y, Suzuki N, Hattori A, Miki H, Yamamura M, Yonenobu K, et al. Hip motion analysis using multi phase (virtual and physical) simulation of the patient-specific hip joint dynamics. Studies in health technology and informatics. 2008;132:339-44.
68. Ogawa H, Kurosaka K, Sato A, Hirasawa N, Matsubara M, Tsukada S. Does An Augmented Reality-based Portable Navigation System Improve the Accuracy of Acetabular Component Orientation During THA? A Randomized Controlled Trial. Clin Orthop Relat Res. 2020;478(5):935-43.
69. Ogawa H, Kurosaka K, Sato A, Hirasawa N, Matsubara M, Tsukada S. Does An Augmented Reality-based Portable Navigation System Improve the Accuracy of Acetabular Component Orientation During THA? A Randomized Controlled Trial. Clinical orthopaedics and related research. 2020;478(5):935‐43.
70. Ogawa H, Kurosaka K, Sato A, Hirasawa N, Matsubara M, Tsukada S. Does An Augmented Reality-based Portable Navigation System Improve the Accuracy of Acetabular Component Orientation during THA? A Randomized Controlled Trial. Clinical Orthopaedics and Related Research. 2020;478(5):935-43.
71. Ogawa H, Kurosaka K, Sato A, Hirasawa N, Matsubara M, Tsukada S. Does An Augmented Reality-based Portable Navigation System Improve the Accuracy of Acetabular Component Orientation During THA? A Randomized Controlled Trial. Clin Orthop Relat Res. 2020;478(5):935-43.
72. Ogawa H, Hasegawa S, Tsukada S, Matsubara M. A Pilot Study of Augmented Reality Technology Applied to the Acetabular Cup Placement During Total Hip Arthroplasty. J Arthroplasty. 2018;33(6):1833-7.
73. Ogawa H, Hasegawa S, Tsukada S, Matsubara M. A Pilot Study of Augmented Reality Technology Applied to the Acetabular Cup Placement During Total Hip Arthroplasty. Journal of arthroplasty. 2018;33(6):1833‐7.
74. Ogawa H, Hasegawa S, Tsukada S, Matsubara M. A Pilot Study of Augmented Reality Technology Applied to the Acetabular Cup Placement During Total Hip Arthroplasty. Journal of Arthroplasty. 2018;33(6):1833-7.
75. Ogawa H, Hasegawa S, Tsukada S, Matsubara M. A Pilot Study of Augmented Reality Technology Applied to the Acetabular Cup Placement During Total Hip Arthroplasty. J Arthroplasty. 2018;33(6):1833-7.
76. Nl. Effectiviteit van Virtual Reality op de pijn en angst beleving na een totale knie of heup prothese operatie. https://trialsearchwhoint/Trial2aspx?TrialID=NL9602. 2021.
77. Nct. Does Virtual Reality Rehabilitation Improve Mobility, Balance, and Walking in Patients With Total Hip Arthroplasty? https://clinicaltrialsgov/show/NCT05173480. 2021.
78. Nct. Does Virtual Reality Rehabilitation Improve Mobility, Balance, and Walking in Patients With Total Hip Arthroplasty? https://clinicaltrialsgov/show/NCT05173480. 2021.
79. Nct. Digital Versus Conventional Physical Therapy for Chronic Shoulder Tendinopathy. https://clinicaltrialsgov/show/NCT04636528. 2020.
80. Nct. Digital Versus Conventional Physical Therapy for Chronic Shoulder Tendinopathy. https://clinicaltrialsgov/show/NCT04636528. 2020.
81. Nct. Early Virtual Reality Based Home Rehabilitation Program After Total Hip Arthroplasty. https://clinicaltrialsgov/show/NCT04221425. 2020.
82. Nct. Early Virtual Reality Based Home Rehabilitation Program After Total Hip Arthroplasty. https://clinicaltrialsgov/show/NCT04221425. 2020.
83. Nct. Effects of Virtual Reality Rehabilitation in Patients With Total Knee Arthroplasty. https://clinicaltrialsgov/show/NCT02413996. 2015.
84. Nct. Effects of Virtual Reality Rehabilitation in Patients With Total Knee Arthroplasty. https://clinicaltrialsgov/show/NCT02413996. 2015.
85. Nakao M, Aso S, Imai Y, Ueda N, Hatanaka T, Shiba M, et al. Automated Planning With Multivariate Shape Descriptors for Fibular Transfer in Mandibular Reconstruction. Ieee Transactions on Biomedical Engineering. 2017;64(8):1772-85.
86. Mishra A, Verma T, Rajkumar, Agarwal G, Sharma A, Maini L. 3D Printed Patient-Specific Acetabular Jig for Cup Placement in Total Hip Arthroplasty. Indian journal of orthopaedics. 2020;54(2):174‐80.
87. Mayoral R, Tsagarakis NG, Petrone M, Clapworthy GJ, Caldwell DG, Zannoni C, et al., editors. Integration of haptic and visual modalities for a total hip replacement planning system. 3rd International Conference on Medical Information Visualisation - BioMedical Visualisation; 2005 2005Jul 05-07; London, ENGLAND2005.
88. Mayoral R, Clapworthy G, editors. Collision detection and free-form deformation for a total hip replacement planning system. 8th Joint Conference on Information Sciences (JCIS 2005); 2005 2005Jul 21-26; Salt Lake City, UT2005.
89. Mao RQ, Lan L, Kay J, De Sa D. Immersive virtual reality (iVR) improves procedural duration, task completion, and accuracy in surgical trainees: a systematic review. CMAJ Canadian Medical Association Journal. 2020;63(6):S71.
90. Manzone M, Airoldi G, Balsari P. Energetic and economic evaluation of a poplar cultivation for the biomass production in Italy. Biomass & Bioenergy. 2009;33(9):1258-64.
91. Logishetty K, Western L, Morgan R, Iranpour F, Cobb JP, Auvinet E. Can an Augmented Reality Headset Improve Accuracy of Acetabular Cup Orientation in Simulated THA? A Randomized Trial. Clin Orthop Relat Res. 2019;477(5):1190-9.
92. Logishetty K, Western L, Morgan R, Iranpour F, Cobb JP, Auvinet E. Can an Augmented Reality Headset Improve Accuracy of Acetabular Cup Orientation in Simulated THA? A Randomized Trial. Clinical orthopaedics and related research. 2019;477(5):1190‐9.
93. Logishetty K, Western L, Morgan R, Iranpour F, Cobb JP, Auvinet E. Can an Augmented Reality Headset Improve Accuracy of Acetabular Cup Orientation in Simulated THA? A Randomized Trial. Clinical Orthopaedics and Related Research. 2019;477(5):1190-9.
94. Logishetty K, Western L, Morgan R, Iranpour F, Cobb JP, Auvinet E. Can an Augmented Reality Headset Improve Accuracy of Acetabular Cup Orientation in Simulated THA? A Randomized Trial. Clin Orthop Relat Res. 2019;477(5):1190-9.
95. Logishetty K, Rudran B, Cobb JP. Virtual reality training improves trainee performance in total hip arthroplasty: a randomized controlled trial. Bone Joint J. 2019;101-b(12):1585-92.
96. Logishetty K, Rudran B, Cobb JP. Virtual reality training improves trainee performance in total hip arthroplasty: a randomized controlled trial. The bone & joint journal. 2019;101‐B(12):1585‐92.
97. Logishetty K, Rudran B, Cobb JP. Virtual reality training improves trainee performance in total hip arthroplasty: a randomized controlled trial. Bone & Joint Journal. 2019;101B(12):1585-92.
98. Logishetty K, Rudran B, Cobb JP. Virtual reality training improves trainee performance in total hip arthroplasty: A randomized controlled trial. Bone and Joint Journal. 2019;101-B(12):1585-92.
99. Logishetty K, Gofton WT, Rudran B, Beaulé PE, Gupte CM, Cobb JP. A Multicenter Randomized Controlled Trial Evaluating the Effectiveness of Cognitive Training for Anterior Approach Total Hip Arthroplasty. J Bone Joint Surg Am. 2020;102(2):e7.
100. Logishetty K, Gofton WT, Rudran B, Beaulé PE, Gupte CM, Cobb JP. A Multicenter Randomized Controlled Trial Evaluating the Effectiveness of Cognitive Training for Anterior Approach Total Hip Arthroplasty. Journal of bone and joint surgery American volume. 2020;102(2):e7.
101. Logishetty K, Gofton WT, Rudran B, Beaule PE, Gupte CM, Cobb JP. A Multicenter Randomized Controlled Trial Evaluating the Effectiveness of Cognitive Training for Anterior Approach Total Hip Arthroplasty. Journal of Bone and Joint Surgery-American Volume. 2020;102(2).
102. Logishetty K, Gofton WT, Rudran B, Beaulé PE, Cobb JP. Fully Immersive Virtual Reality for Total Hip Arthroplasty: Objective Measurement of Skills and Transfer of Visuospatial Performance After a Competency-Based Simulation Curriculum. J Bone Joint Surg Am. 2020;102(6):e27.
103. Logishetty K, Gofton WT, Rudran B, Beaulé PE, Cobb JP. Fully Immersive Virtual Reality for Total Hip Arthroplasty: Objective Measurement of Skills and Transfer of Visuospatial Performance after a Competency-Based Simulation Curriculum. Journal of Bone and Joint Surgery - American Volume. 2020;102(6).
104. Logishetty K, Gofton WT, Rudran B, Beaule PE, Cobb JP. Fully Immersive Virtual Reality for Total Hip Arthroplasty: Objective Measurement of Skills and Transfer of Visuospatial Performance After a Competency-Based Simulation Curriculum. Journal of Bone and Joint Surgery-American Volume. 2020;102(6).
105. Liu H, Auvinet E, Giles J, Baena FRy. Augmented Reality Based Navigation for Computer Assisted Hip Resurfacing: A Proof of Concept Study. Annals of biomedical engineering. 2018;46(10):1595-605.
106. Leopold SS. Editor's Spotlight/Take 5: Does An Augmented Reality-based Portable Navigation System Improve the Accuracy of Acetabular Component Orientation During THA? A Randomized Controlled Trial. Clin Orthop Relat Res. 2020;478(5):931-4.
107. Leopold SS. Editor's Spotlight/Take 5: does An Augmented Reality-based Portable Navigation System Improve the Accuracy of Acetabular Component Orientation during THA? A Randomized Controlled Trial. Clinical orthopaedics and related research. 2020;478(5):931‐4.
108. Leopold SS. Editor's Spotlight/Take 5: Does An Augmented Reality-based Portable Navigation System Improve the Accuracy of Acetabular Component Orientation during THA? A Randomized Controlled Trial. Clinical Orthopaedics and Related Research. 2020;478(5):931-4.
109. Leopold SS. Editor's Spotlight/Take 5: Does An Augmented Reality-based Portable Navigation System Improve the Accuracy of Acetabular Component Orientation During THA? A Randomized Controlled Trial. Clin Orthop Relat Res. 2020;478(5):931-4.
110. Leng J, Al-Hajjar M, Wilcox R, Jones A, Barton D, Fisher J. Dynamic virtual simulation of the occurrence and severity of edge loading in hip replacements associated with variation in the rotational and translational surgical position. Proceedings of the Institution of Mechanical Engineers Part H-Journal of Engineering in Medicine. 2017;231(4):299-306.
111. Lei P-f, Su S-l, Kong L-y, Wang C-g, Zhong D, Hu Y-h. Mixed Reality Combined with Three-Dimensional Printing Technology in Total Hip Arthroplasty: An Updated Review with a Preliminary Case Presentation. Orthopaedic Surgery. 2019;11(5):914-20.
112. Lei PF, Su SL, Kong LY, Wang CG, Zhong D, Hu YH. Mixed Reality Combined with Three-Dimensional Printing Technology in Total Hip Arthroplasty: An Updated Review with a Preliminary Case Presentation. Orthop Surg. 2019;11(5):914-20.
113. Lei PF, Su SL, Kong LY, Wang CG, Zhong D, Hu YH. Mixed Reality Combined with Three-Dimensional Printing Technology in Total Hip Arthroplasty: An Updated Review with a Preliminary Case Presentation. Orthopaedic Surgery. 2019;11(5):914-20.
114. Łęgosz P, Starszak K, Stanuch M, Otworowski M, Pulik Ł, Złahoda-Huzior A, et al. The Use of Mixed Reality in Custom-Made Revision Hip Arthroplasty: A First Case Report. Journal of visualized experiments : JoVE. 2022(186).
115. Legosz P, Starszak K, Stanuch M, Otworowski M, Pulik L, Zlahoda-Huzior A, et al. The Use of Mixed Reality in Custom-Made Revision Hip Arthroplasty: A First Case Report. Journal of visualized experiments : JoVE. 2022(186).
116. Ledford CK, VanWagner MJ, Sherman CE, Torp KD. Immersive Virtual Reality Used as Adjunct Anesthesia for Conversion Total Hip Arthroplasty in a 100-Year-Old Patient. Arthroplasty Today. 2021;10:149-53.
117. Ledford CK, VanWagner MJ, Sherman CE, Torp KD. Immersive Virtual Reality Used as Adjunct Anesthesia for Conversion Total Hip Arthroplasty in a 100-Year-Old Patient. Arthroplasty Today. 2021;10:149-53.
118. Krokos M, Podgorelec D, Clapworthy GJ, Liang RH, Testi D, Viceconti M, et al., editors. Patient-specific muscle models for surgical planning. 3rd International Conference on Medical Information Visualisation - BioMedical Visualisation; 2005 2005Jul 05-07; London, ENGLAND2005.
119. Krause L, Farrow D, Pinder R, Buszard T, Kovalchik S, Reid M. Enhancing skill transfer in tennis using representative learning design. Journal of sports sciences. 2019;37(22):2560‐8.
120. Kobayashi S, Cho B, Huaulme A, Tatsugami K, Honda H, Jannin P, et al. Assessment of surgical skills by using surgical navigation in robot-assisted partial nephrectomy. International journal of computer assisted radiology and surgery. 2019;14(8):1449-59.
121. Khodarahmi I, Fritz J. The Value of 3 Tesla Field Strength for Musculoskeletal Magnetic Resonance Imaging. Investigative Radiology. 2021;56(11):749-63.
122. Keating TC, Jacobs JJ. Augmented Reality in Orthopedic Practice and Education. Orthop Clin North Am. 2021;52(1):15-26.
123. Keating TC, Jacobs JJ. Augmented Reality in Orthopedic Practice and Education. Orthopedic Clinics of North America. 2021;52(1):15-26.
124. Keating TC, Jacobs JJ. Augmented Reality in Orthopedic Practice and Education. Orthop Clin North Am. 2021;52(1):15-26.
125. Kaluschke M, Weller R, Zachmann G, Pelliccia L, Lorenz M, Klimant P, et al., editors. HIPS - A Virtual Reality Hip Prosthesis Implantation Simulator. 25th IEEE Conference on Virtual Reality and 3D User Interfaces (IEEE VR); 2018 2018Mar 18-22; Reutlingen, GERMANY2018.
126. Joshi R, Joseph A, Mihandoust S, Madathil KC, Cotten SR. A mobile application-based home assessment tool for patients undergoing joint replacement surgery: A qualitative feasibility study. Applied ergonomics. 2022;103:103796.
127. Joshi R, Joseph A, Mihandoust S, Madathil KC, Cotten SR. A mobile application-based home assessment tool for patients undergoing joint replacement surgery: A qualitative feasibility study. Applied ergonomics. 2022;103.
128. Joshi R, Joseph A, Mihandoust S, Madathil KC, Cotten SR. A mobile application-based home assessment tool for patients undergoing joint replacement surgery: A qualitative feasibility study. Applied ergonomics. 2022;103:103796.
129. Isrctn. Is learning together in virtual reality better than learning individually for surgical teams training for complex operations? https://trialsearchwhoint/Trial2aspx?TrialID=ISRCTN32225943. 2022.
130. Isrctn. Virtual reality training for hip surgery. https://trialsearchwhoint/Trial2aspx?TrialID=ISRCTN13074978. 2018.
131. Imboden S, Petrone M, Quadrani P, Zannoni C, Mayoral R, Clapworthy GJ, et al., editors. A haptic enabled multimodal pre-operative planner for hip arthroplasty. 1st Joint Eurohaptics Conference/Symposium on Haptic Interfaces for Virtual Environment and Teleoperator Systems; 2005 2005Mar 18-20; Pisa, ITALY2005.
132. Huang MY, Scharf S, Chan PY. Effects of immersive virtual reality therapy on intravenous patient-controlled sedation during orthopaedic surgery under regional anesthesia: a randomized controlled trial. PloS one. 2020;15(2):e0229320.
133. Huang MY, Scharf S, Chan PY. Effects of immersive virtual reality therapy on intravenous patient-controlled sedation during orthopaedic surgery under regional anesthesia: A randomized controlled trial. PLoS ONE. 2020;15(2).
134. Huang MY, Scharf S, Chan PY. Effects of immersive virtual reality therapy on intravenous patient-controlled sedation during orthopaedic surgery under regional anesthesia: a randomized controlled trial. PloS one. 2020;15(2):e0229320.
135. Hooper J, Tsiridis E, Feng JE, Schwarzkopf R, Waren D, Long WJ, et al. Virtual Reality Simulation Facilitates Resident Training in Total Hip Arthroplasty: A Randomized Controlled Trial. Journal of Arthroplasty. 2019;34(10):2278-83.
136. Hooper J, Tsiridis E, Feng JE, Schwarzkopf R, Waren D, Long WJ, et al. Virtual Reality Simulation Facilitates Resident Training in Total Hip Arthroplasty: A Randomized Controlled Trial. Journal of Arthroplasty. 2019;34(10):2278-83.
137. Hooper J, Tsiridis E, Feng JE, Schwarzkopf R, Waren D, Long WJ, et al. Virtual Reality Simulation Facilitates Resident Training in Total Hip Arthroplasty: A Randomized Controlled Trial. J Arthroplasty. 2019;34(10):2278-83.
138. Hooper J, Tsiridis E, Feng JE, Schwarzkopf R, Waren D, Long WJ, et al. Virtual Reality Simulation Facilitates Resident Training in Total Hip Arthroplasty: a Randomized Controlled Trial. Journal of arthroplasty. 2019;34(10):2278‐83.
139. Holt G, Nunn T, Gregori A. Ethical Dilemmas in Orthopedic Surgical Training. Journal of Bone and Joint Surgery-American Volume. 2008;90A(12):2798-803.
140. Handels H, Ehrhardt J, Plotz W, Poppl SJ. Three-dimensional planning and simulation of hip operations and computer-assisted construction of endoprostheses in bone tumor surgery. Computer aided surgery : official journal of the International Society for Computer Aided Surgery. 2001;6(2):65-76.
141. Handels H, Ehrhardt J, Plotz W, Poppl SJ. Simulation of hip operations and design of custom-made endoprostheses using virtual reality techniques. Methods of Information in Medicine. 2001;40(2):74-7.
142. Handels H, Ehrhardt J, Plotz W, Poppl SJ. Virtual planning of hip operations and individual adaption of endoprostheses in orthopaedic surgery. International Journal of Medical Informatics. 2000;58:21-8.
143. Haluzynskyi OA, Chornyi VS, Burburska SV, Kozik YV. USE OF COMPUTER NAVIGATION IN TOTAL HIP ARTHROPLASTY (LITERATURE REVIEW). Wiadomosci lekarskie (Warsaw, Poland : 1960). 2022;75(6):1765-70.
144. Haluzynskyi OA, Chornyi VS, Burburska SV, Kozik YV. USE OF COMPUTER NAVIGATION IN TOTAL HIP ARTHROPLASTY (LITERATURE REVIEW). Wiadomosci lekarskie (Warsaw, Poland : 1960). 2022;75(6):1765-70.
145. Haluzynskyi OA, Chornyi VS, Burburska SV, Kozik YV. USE OF COMPUTER NAVIGATION IN TOTAL HIP ARTHROPLASTY (LITERATURE REVIEW). Wiadomosci lekarskie (Warsaw, Poland : 1960). 2022;75(6):1765-70.
146. Hall AJ, Walmsley P. Technology-enhanced learning in orthopaedics: Virtual reality and multi-modality educational workshops may be effective in the training of surgeons and operating department staff. The surgeon : journal of the Royal Colleges of Surgeons of Edinburgh and Ireland. 2022.
147. Halawi MJ, Cote MP, Singh H, O'Sullivan MB, Savoy L, Lieberman JR, et al. The Effect of Depression on Patient-Reported Outcomes After Total Joint Arthroplasty Is Modulated by Baseline Mental Health: A Registry Study. J Bone Joint Surg Am. 2018;100(20):1735-41.
148. Halawi MJ, Cote MP, Singh H, O'Sullivan MB, Savoy L, Lieberman JR, et al. The Effect of Depression on Patient-Reported Outcomes After Total Joint Arthroplasty Is Modulated by Baseline Mental Health A Registry Study. Journal of Bone and Joint Surgery-American Volume. 2018;100(20):1735-41.
149. Hadamus A, Bialoszewski D, Wydra K, Kowalska AJ, Urbaniak E, Boratyński R, et al. Balance training in virtual reality improves temporal gait parameters in patients after total hip replacement. Gait and Posture. 2019;73:99.
150. Hadamus A, Bialoszewski D, Wydra K, Kowalska AJ, Urbaniak E, Boratynski R, et al. Balance training in virtual reality improves temporal gait parameters in patients after total hip replacement. Gait & posture. 2019;73:99‐.
151. Hadamus A, Białoszewski D, Urbaniak E, Wydra K, Kowalska AJ, Boratyński R, et al. Balance training in virtual reality in patients after hip replacement does not influence foot pressure distribution in static balance test. Gait and Posture. 2019;73:272.
152. Hadamus A, Bialoszewski D, Urbaniak E, Wydra K, Kowalska AJ, Boratynski R, et al. Balance training in virtual reality in patients after hip replacement does not influence foot pressure distribution in static balance test. Gait & posture. 2019;73:272‐.
153. Hadamus A, Białoszewski D, Urbaniak E, Kowalska AJ, Wydra K, Boratyński R, et al. The impact of training in virtual reality on balance in patients after total knee replacement is relatively slight. Gait and Posture. 2020;81:134-5.
154. Hadamus A, Białoszewski D, Kowalska AJ, Urbaniak E, Wydra K, Boratyński R, et al. There is little impact of balance training in Virtual Reality on selected balance parameters in patients after hip arthroplasty. Gait and Posture. 2019;73:588.
155. Hadamus A, Bialoszewski D, Kowalska AJ, Urbaniak E, Wydra K, Boratynski R, et al. There is little impact of balance training in Virtual Reality on selected balance parameters in patients after hip arthroplasty. Gait & posture. 2019;73:588‐.
156. Hadamus A, Białoszewski D, Kowalska AJ, Urbaniak E, Boratynski R, Marczynski W. Impact of balance training in virtual reality on body balance in patients after total hip replacement. Age and Ageing. 2019;48(2):iv28.
157. Hadamus A, Bialoszewski D, Kowalska AJ, Urbaniak E, Boratynski R, Marczynski W. Impact of balance training in virtual reality on body balance in patients after total hip replacement. Age and ageing. 2019;48(2):iv28‐.
158. Guggenberger B, Jocham AJ, Jocham B, Nischelwitzer A, Ritschl H. Instrumental Validity of the Motion Detection Accuracy of a Smartphone-Based Training Game. International journal of environmental research and public health. 2021;18(16).
159. Guggenberger B, Jocham AJ, Jocham B, Nischelwitzer A, Ritschl H. Instrumental validity of the motion detection accuracy of a smartphone-based training game. International journal of environmental research and public health. 2021;18(16).
160. Guggenberger B, Jocham AJ, Jocham B, Nischelwitzer A, Ritschl H. Instrumental Validity of the Motion Detection Accuracy of a Smartphone-Based Training Game. International journal of environmental research and public health. 2021;18(16).
161. Gianola S, Stucovitz E, Castellini G, Mascali M, Vanni F, Tramacere I, et al. Effects of early virtual reality-based rehabilitation in patients with total knee arthroplasty: A randomized controlled trial. Medicine. 2020;99(7).
162. Fotouhi J, Unberath M, Song T, Gu W, Johnson A, Osgood G, et al. Interactive Flying Frustums (IFFs): spatially aware surgical data visualization. International journal of computer assisted radiology and surgery. 2019;14(6):913-22.
163. Fotouhi J, Unberath M, Song T, Gu W, Johnson A, Osgood G, et al. Interactive Flying Frustums (IFFs): spatially aware surgical data visualization. International journal of computer assisted radiology and surgery. 2019;14(6):913-22.
164. Fotouhi J, Unberath M, Song T, Gu W, Johnson A, Osgood G, et al. Interactive Flying Frustums (IFFs): spatially aware surgical data visualization. International journal of computer assisted radiology and surgery. 2019.
165. Fotouhi J, Mehrfard A, Song T, Johnson A, Osgood G, Unberath M, et al. Development and Pre-Clinical Analysis of Spatiotemporal-Aware Augmented Reality in Orthopedic Interventions. IEEE transactions on medical imaging. 2021;40(2):765-78.
166. Fotouhi J, Mehrfard A, Song T, Johnson A, Osgood G, Unberath M, et al. Development and Pre-Clinical Analysis of Spatiotemporal-Aware Augmented Reality in Orthopedic Interventions. IEEE transactions on medical imaging. 2021;40(2):765-78.
167. Fotouhi J, Mehrfard A, Song T, Johnson A, Osgood G, Unberath M, et al. Development and Pre-Clinical Analysis of Spatiotemporal-Aware Augmented Reality in Orthopedic Interventions. IEEE transactions on medical imaging. 2021;40(2):765-78.
168. Fotouhi J, Alexander CP, Unberath M, Taylor G, Lee SC, Fuerst B, et al., editors. Technical Note: An Augmented Reality System for Total Hip Arthroplasty. Conference on Medical Imaging - Image-Guided Procedures, Robotic Interventions, and Modeling; 2018 2018Feb 12-15; Houston, TX2018.
169. Fotouhi J, Alexander CP, Unberath M, Taylor G, Lee SC, Fuerst B, et al. Plan in 2-D, execute in 3-D: an augmented reality solution for cup placement in total hip arthroplasty. Journal of medical imaging (Bellingham, Wash). 2018;5(2):021205.
170. Fotouhi J, Alexander CP, Unberath M, Taylor G, Lee SC, Fuerst B, et al. Plan in 2-D, execute in 3-D: An augmented reality solution for cup placement in total hip arthroplasty. Journal of Medical Imaging. 2018;5(2).
171. Fotouhi J, Alexander CP, Unberath M, Taylor G, Lee SC, Fuerst B, et al. Plan in 2-D, execute in 3-D: an augmented reality solution for cup placement in total hip arthroplasty. Journal of medical imaging (Bellingham, Wash). 2018;5(2):021205.
172. Fontalis A, Epinette J-A, Thaler M, Zagra L, Khanduja V, Haddad FS. Advances and innovations in total hip arthroplasty. Sicot-J. 2021;7.
173. Favre P, Maquer G, Henderson A, Hertig D, Ciric D, Bischoff JE. In Silico Clinical Trials in the Orthopedic Device Industry: From Fantasy to Reality? Annals of biomedical engineering. 2021;49(12):3213-26.
174. Fascio E, Vitale JA, Sirtori P, Peretti G, Banfi G, Mangiavini L. Early Virtual-Reality-Based Home Rehabilitation after Total Hip Arthroplasty: A Randomized Controlled Trial. J Clin Med. 2022;11(7).
175. Fascio E, Vitale JA, Sirtori P, Peretti G, Banfi G, Mangiavini L. Early Virtual-Reality-Based Home Rehabilitation after Total Hip Arthroplasty: A Randomized Controlled Trial. Journal of Clinical Medicine. 2022;11(7).
176. Fascio E, Vitale JA, Sirtori P, Peretti G, Banfi G, Mangiavini L. Early Virtual-Reality-Based Home Rehabilitation after Total Hip Arthroplasty: A Randomized Controlled Trial. J Clin Med. 2022;11(7).
177. Fascio E, Vitale JA, Sirtori P, Peretti G, Banfi G, Mangiavini L. Early Virtual-Reality-Based Home Rehabilitation after Total Hip Arthroplasty: A Randomized Controlled Trial. J Clin Med. 2022;11(7).
178. Edwards T, Gupta S, Soussi D, Patel A, Khan S, Liddle A, et al. Collaborative Surgical Team Training in Virtual Reality Is Superior to Individual Learning: A Randomised Controlled Trial. British Journal of Surgery. 2022;109:vi10.
179. Duke JN, Yang C. Advances in instrumentation and enhancing technology for direct anterior hip replacement surgery. Annals of Joint. 2018;3(5).
180. De Boey S, Maes M, Mertens P. Teaching hip surgery to orthopaedic residents: what's new? Hip Int. 2020;30(1_suppl):42-7.
181. De Boey S, Maes M, Mertens P. Teaching hip surgery to orthopaedic residents: what's new? Hip International. 2020;30(1_SUPPL):42-7.
182. Cutolo F. Letter to the Editor on "Augmented Reality Based Navigation for Computer Assisted Hip Resurfacing: A Proof of Concept Study". Annals of biomedical engineering. 2019;47(11):2151-3.
183. Cutolo F. Letter to the Editor on "Augmented Reality Based Navigation for Computer Assisted Hip Resurfacing: A Proof of Concept Study". Annals of biomedical engineering. 2019;47(11):2151-3.
184. Condino S, Turini G, Parchi PD, Viglialoro RM, Piolanti N, Gesi M, et al. How to Build a Patient-Specific Hybrid Simulator for Orthopaedic Open Surgery: Benefits and Limits of Mixed-Reality Using the Microsoft HoloLens. J Healthc Eng. 2018;2018:5435097.
185. Condino S, Turini G, Parchi PD, Viglialoro RM, Piolanti N, Gesi M, et al. How to Build a Patient-Specific Hybrid Simulator for Orthopaedic Open Surgery: Benefits and Limits of Mixed-Reality Using the Microsoft HoloLens. Journal of Healthcare Engineering. 2018;2018.
186. Cleary K, Peters TM. Image-Guided Interventions: Technology Review and Clinical Applications. In: Yarmush ML, Duncan JS, Gray ML, editors. Annual Review of Biomedical Engineering, Vol 12. Annual Review of Biomedical Engineering. 122010. p. 119-42.
187. Clapworthy GJ, Krokos M, Mayoral R, Liang R, Podgorelec D, editors. Visualisation within a multisensorial surgical planner. 2nd International Conference on Computer Graphics, Imaging and Vision (CGVIS 2005); 2005 2005Jul 26-29; Beijing, PEOPLES R CHINA2005.
188. Chughtai M, Kelly JJ, Newman JM, Sultan AA, Khlopas A, Sodhi N, et al. The Role of Virtual Rehabilitation in Total and Unicompartmental Knee Arthroplasty. Journal of Knee Surgery. 2019;32(1):105-10.
189. Cho HK, Sunghoon K. A Study on Affordance Design Characteristics in Augmented Reality(AR) Digital Signage Advertisement. Journal of the Korean Society of Design Culture. 2018;24(3):611-23.
190. ChiCtr. The influence of early rehabilitation based on early virtual reality-based rehabilitation on patients with THA or TKA. http://wwwwhoint/trialsearch/Trial2aspx?TrialID=ChiCTR2000038292. 2020.
191. ChiCtr. Safety and efficacy of mixed reality in complex artificial joint replacement. http://wwwwhoint/trialsearch/Trial2aspx?TrialID=ChiCTR1900026133. 2019.
192. ChiCtr. Safety and efficacy of mixed reality combined with 3D printing instrument in complex artificial joint replacement. http://wwwwhoint/trialsearch/Trial2aspx?TrialID=ChiCTR1900026119. 2019.
193. Byra J, Czernicki K. The Effectiveness of Virtual Reality Rehabilitation in Patients with Knee and Hip Osteoarthritis. Journal of Clinical Medicine. 2020;9(8).
194. Byra J, Czernicki K. The effectiveness of virtual reality rehabilitation in patients with knee and hip osteoarthritis. Journal of Clinical Medicine. 2020;9(8):1-18.
195. Brumat P, Kunsic O, Novak S, Slokar U, Psenica J, Topolovec M, et al. The Surgical Treatment of Osteoarthritis. Life-Basel. 2022;12(7).
196. Blyth P, Stott NS, Anderson IA. Virtual reality assessment of technical skill using the Bonedoc DHS simulator. Injury-International Journal of the Care of the Injured. 2008;39(10):1127-33.
197. Blasco JM, Igual-Camacho C, Blasco MC, Anton-Anton V, Ortiz-Llueca A, Roig-Casasus S. The efficacy of virtual reality tools for total knee replacement rehabilitation: A systematic review. Physiotherapy Theory and Practice. 2021;37(6):682-92.
198. Blanco PJ, Holliman RP, Ceballos PL, Farnam JL. Exploring the Impact of Child-Centered Play Therapy on Academic Achievement of At-Risk Kindergarten Students. International journal of play therapy. 2019;28(3):133‐43.
199. Berton A, Longo UG, Candela V, Fioravanti S, Giannone L, Arcangeli V, et al. Virtual Reality, Augmented Reality, Gamification, and Telerehabilitation: Psychological Impact on Orthopedic Patients' Rehabilitation. Journal of Clinical Medicine. 2020;9(8).
200. Barratt H, Turner S, Hutchings A, Pizzo E, Hudson E, Briggs T, et al. Mixed methods evaluation of the Getting it Right First Time programme - improvements to NHS orthopaedic care in England: study protocol. BMC health services research. 2017;17(1):71.
201. Barratt H, Turner S, Hutchings A, Pizzo E, Hudson E, Briggs T, et al. Mixed methods evaluation of the Getting it Right First Time programme - improvements to NHS orthopaedic care in England: study protocol. BMC health services research. 2017;17.
202. Barrack RL, Lavernia C, Ries M, Thornberry R, Tozakoglou E. Virtual reality computer animation of the effect of component position and design on stability after total hip arthroplasty. Orthopedic Clinics of North America. 2001;32(4):569-+.
203. Barrack RL, Lavernia C, Ries M, Thornberry R, Tozakoglou E. Virtual reality computer animation of the effect of component position and design on stability after total hip arthroplasty. Orthopedic Clinics of North America. 2001;32(4):569-77.
204. Barrack RL, Butler RA, Laster DR, Andrews P. Stem design and dislocation after revision total hip arthroplasty - Clinical results and computer modeling. Journal of Arthroplasty. 2001;16(8):8-12.
205. Barrack RL, Butler RA, Laster DR, Andrews P. Stem design and dislocation after revision total hip arthroplasty: Clinical results and computer modeling. Journal of Arthroplasty. 2001;16(8 SUPPL. 1):8-12.
206. Bah MT, Shi J, Browne M, Suchier Y, Lefebvre F, Young P, et al. Exploring inter-subject anatomic variability using a population of patient-specific femurs and a statistical shape and intensity model. Medical Engineering & Physics. 2015;37(10):995-1007.
207. Anton D, Nelson M, Russell T, Goni A, Illarramendi A. Validation of a Kinect-based telerehabilitation system with total hip replacement patients. Journal of Telemedicine and Telecare. 2016;22(3):192-7.
208. Anton D, Berges I, Bermudez J, Goni A, Illarramendi A. A Telerehabilitation System for the Selection, Evaluation and Remote Management of Therapies. Sensors. 2018;18(5).
209. Alexander C, Loeb AE, Fotouhi J, Navab N, Armand M, Khanuja HS. Augmented Reality for Acetabular Component Placement in Direct Anterior Total Hip Arthroplasty. J Arthroplasty. 2020;35(6):1636-41.e3.
210. Alexander C, Loeb AE, Fotouhi J, Navab N, Armand M, Khanuja HS. Augmented Reality for Acetabular Component Placement in Direct Anterior Total Hip Arthroplasty. Journal of Arthroplasty. 2020;35(6):1636-+.
211. Alexander C, Loeb AE, Fotouhi J, Navab N, Armand M, Khanuja HS. Augmented Reality for Acetabular Component Placement in Direct Anterior Total Hip Arthroplasty. Journal of Arthroplasty. 2020;35(6):1636-41.e3.
212. Actrn. Telerehabilitation with virtual reality in spastic hemiplegia cerebral palsy. https://trialsearchwhoint/Trial2aspx?TrialID=ACTRN12621000117819. 2021.
213. Actrn. Effectiveness of virtual reality in functionality when used in physiotherapeutic training of patients with total hip arthroplasty compared to conventional treatment. Randomized Clinical Trial. https://trialsearchwhoint/Trial2aspx?TrialID=ACTRN12618001252202. 2018.
