# Supplementary material for: The effectiveness of virtual reality, augmented reality, and mixed reality training in total hip arthroplasty: a systematic review and meta-analysis
Source: J Orthop Surg Res. 2023 Feb 19;18:121. doi: 10.1186/s13018-023-03604-z (PMC9940416; doi:10.1186/s13018-023-03604-z)
Supplement: Supplementary file 3 — Additional file 3. Funnel plots to assess publication bias on the accuracy of inclination and anteversion. [file 13018_2023_3604_MOESM3_ESM.docx]

**Funnel plots to assess publication bias on the accuracy of inclination and anteversion.**


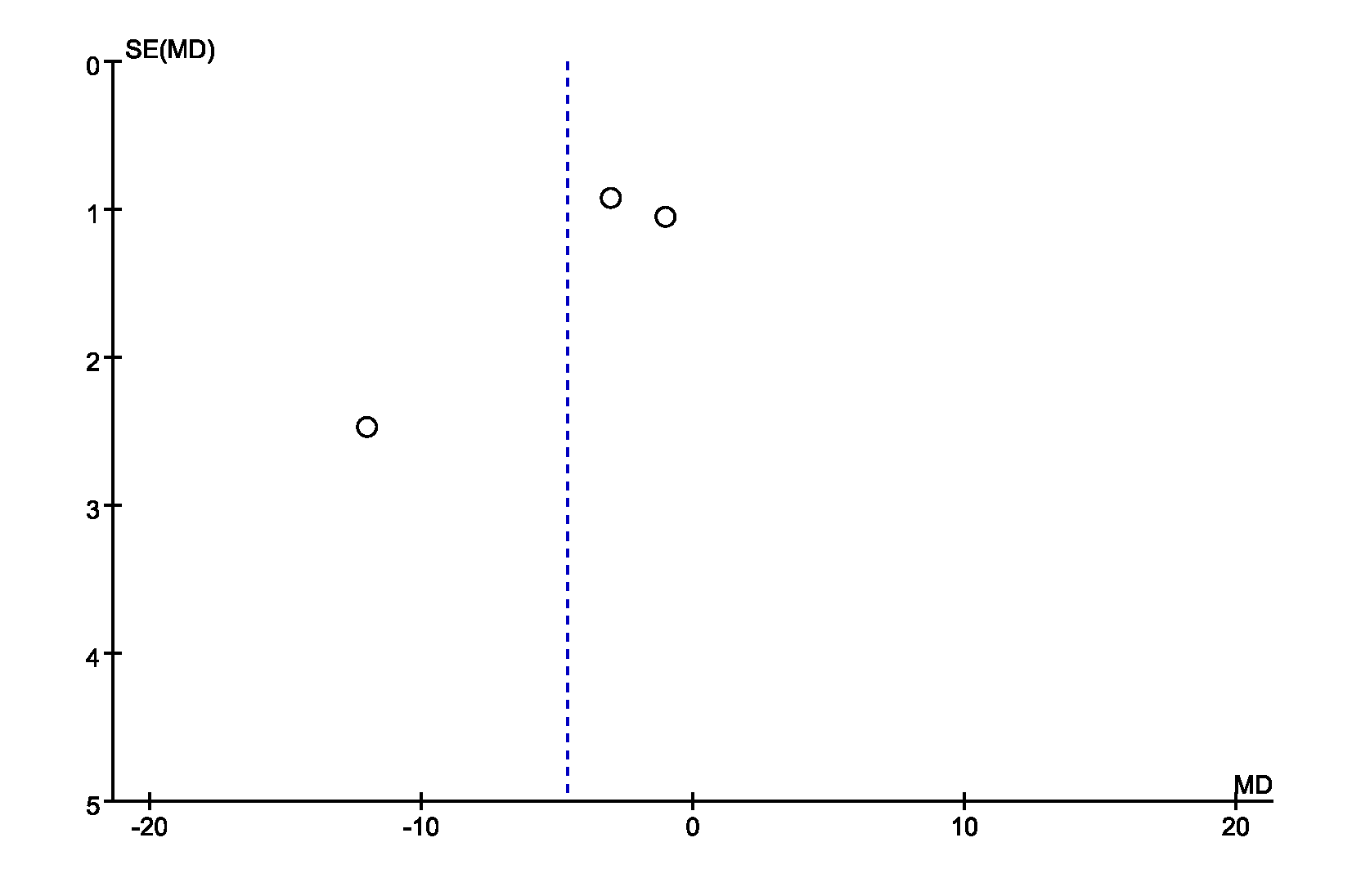


**Figure 1**. Funnel plot to assess publication bias on the accuracy of inclination.


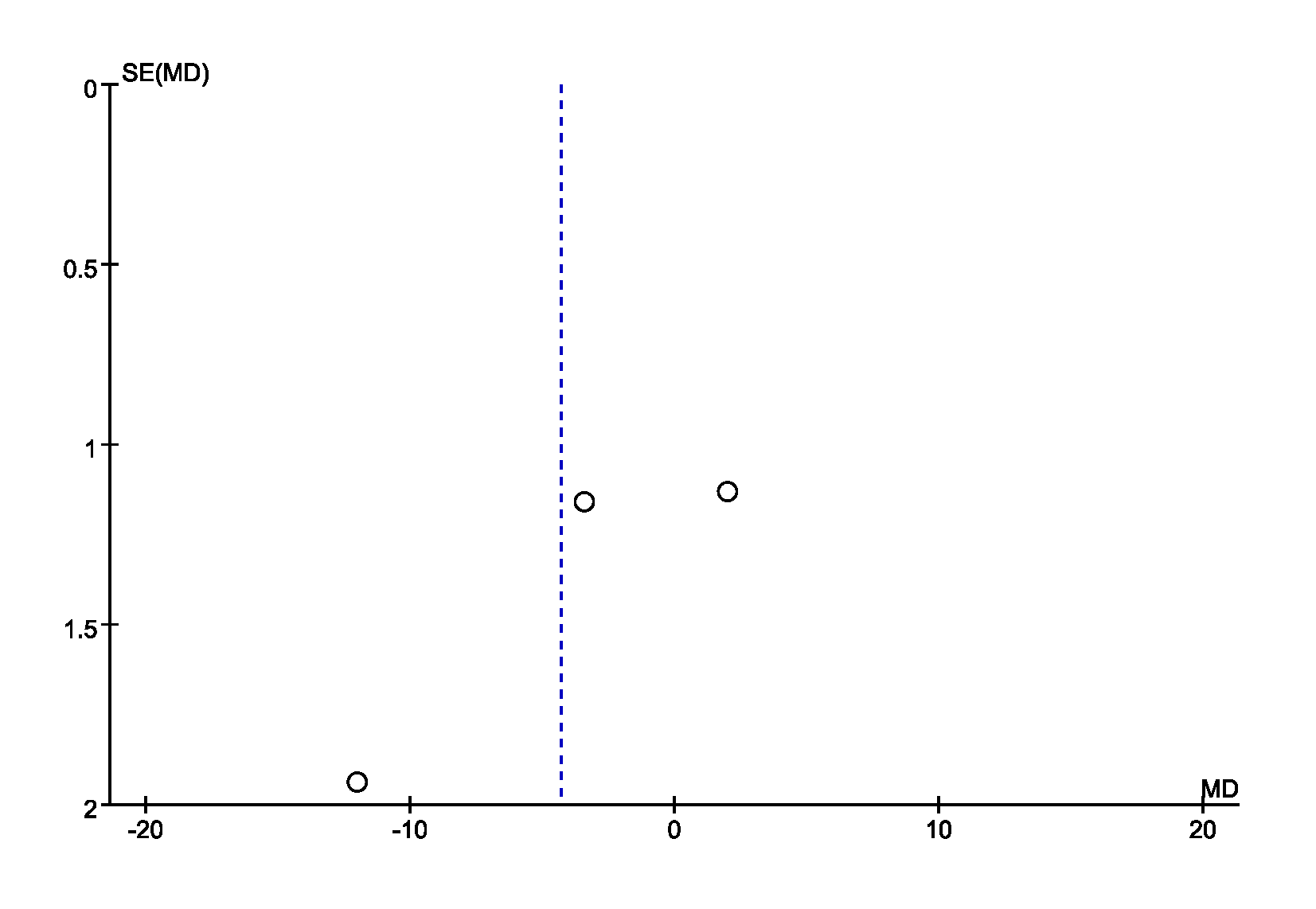


**Figure 2**. Funnel plot to assess publication bias on the accuracy of anteversion.
